# A Novel Human scFv Library with Non-Combinatorial Synthetic CDR Diversity

**DOI:** 10.1371/journal.pone.0141045

**Published:** 2015-10-20

**Authors:** Xuelian Bai, Jihye Kim, Seungmin Kang, Wankyu Kim, Hyunbo Shim

**Affiliations:** 1 Department of Life Science, Ewha Womans University, Seoul, Korea; 2 Department of Bioinspired Science, Ewha Womans University, Seoul, Korea; University of Edinburgh, UNITED KINGDOM

## Abstract

The present work describes the construction and validation of a human scFv library with a novel design approach to synthetic complementarity determining region (CDR) diversification. The advantage of synthetic antibody libraries includes the possibility of exerting fine control over factors like framework sequences, amino acid and codon usage, and CDR diversity. However, random combinatorial synthesis of oligonucleotides for CDR sequence diversity also produces many clones with unnatural sequences and/or undesirable modification motifs. To alleviate these issues, we designed and constructed a novel semi-synthetic human scFv library with non-combinatorial, pre-designed CDR diversity and a single native human framework each for heavy, kappa, and lambda chain variable domains. Next-generation sequencing analysis indicated that the library consists of antibody clones with highly nature-like CDR sequences and the occurrence of the post-translational modification motifs is minimized. Multiple unique clones with nanomolar affinity could be isolated from the library against a number of target antigens, validating the library design strategy. The results demonstrate that it is possible to construct a functional antibody library using low, non-combinatorial synthetic CDR diversity, and provides a new strategy for the design of antibody libraries suitable for demanding applications.

## Introduction

Target-specific antibodies can be rapidly isolated from a large antibody library by *in vitro* display technologies, such as phage or yeast display. The size and quality of the antibody library is a major determinant of the success of *in vitro* antibody generation, and many different strategies have been employed to design and construct large, highly functional antibody libraries [1]. While the size of an antibody library is mostly determined by the transformation efficiency of bacteria or yeast, multiple different factors can influence the functionality of a library and thus need to be considered in the library design. One important factor in library design is the source and nature of the sequence diversity, which can originate from natural (animal B-cells), synthetic, or semi-synthetic sources.

Antibody libraries from natural sources consist of antibody clones with variable regions that are the end product of V(D)J recombination and somatic hypermutation of germline immunoglobulin genes, all of which are presumably optimized through the evolutionary history of adaptive immunity. Antibodies isolated from a natural antibody library are thus generally considered more nature-like than those from a synthetic antibody library, whose diversity typically is generated by random combinatorial events. On the other hand, the greater heterogeneity of the framework regions among the clones of natural antibody libraries may result in more uneven propagation of the clones during the amplification phase of biopanning. Also, somatic hypermutations, especially in the framework regions, may introduce immunogenic sequences that can elicit human anti-human antibody (HAHA) response when the antibody is used as a therapeutic agent.

Synthetic antibody libraries usually have one or a small number of framework sequences, upon which artificially designed and synthesized CDR sequences are grafted. The CDR diversity is mostly generated by concatenating random nucleotides [2–4] or trinucleotide units [5–7]. Various CDR design strategies have been employed to emulate natural CDRs. However, the random combinatorial nature of synthetic CDR design inevitably introduces some non-natural sequences to the library. Conversely, the synthetic library can avoid or reduce many of the problems of the natural antibody library described above by employing framework regions that are fewer in number and so more homogeneous, have germline sequences without mutations, and are chosen for their desirable properties such as stability, solubility and expression level.

In both natural and synthetic antibody repertoires, clones with undesirable post-translational modification (PTM) motifs may exist. These PTMs include *N*-linked glycosylation, aspartate isomerization, asparagine deamidation, non-enzymatic cleavage of peptide backbone, and oxidation of amino acid side chains. These PTM motifs generally do not cause many problems when they occur in natural immunoglobulins produced by B-cells *in vivo*, because of the polyclonality and the continuous turnover of serum antibodies. However, for recombinant antibodies, especially therapeutic antibodies, that are produced and purified in large quantity and stored for extended periods of time, these slow, spontaneous, and non-enzymatic modifications (except *N*-linked glycosylation) may adversely affect the affinity, specificity, and function of the antibodies [8]. It is not practical to eliminate all potential PTM motifs from the library. However, efforts have been made to reduce or minimize certain problematic sequence motifs in the synthetic antibody library. Recently, human Fab libraries from which potential PTM sites were eliminated or minimized have been reported. For example, a Fab library was constructed with omission of asparagines at critical positions to eliminate *N*-glycosylation motifs [6]. In another example [9], potential PTM sites were removed altogether from CDR1s and 2s, and the modification-prone amino acids, such as asparagine, methionine, and cysteine, were omitted or minimized from CDR3s. One potential drawback of this approach is that some of the PTM-prone amino acids like aspartate and asparagine are also among those that are more frequently utilized in the CDRs [10–12].

In this study, a novel semi-synthetic human scFv library was designed and constructed, and its functionality was verified by isolating and analyzing antibodies from the library against a number of antigens, as well as by next generation sequencing analysis. The library was designed to have predetermined CDR sequences with non-combinatorial diversity that dutifully mimic natural CDRs with somatic hypermutations but without many of potential PTM-prone motifs. The diversity of each of the six CDRs is low (~10^3^ unique sequences per each region), but they can combine to make a large library with high functionality. Our results suggest that non-combinatorial synthetic CDR diversity design is a viable approach to the construction of antibody libraries, and that the design principle may have certain advantages that include highly nature-like sequences and the avoidance of undesirable sequence motifs.

## Materials and Methods

### Design of CDR sequences

Human antibody variable region sequences were downloaded from the immunogenetics (IMGT) database (http://imgt.org). CDR sequences were extracted from the sequences according to Kabat CDR definition [13]. Human germline CDR sequences were obtained from V-Base (http://www2.mrc-lmb.cam.ac.uk/vbase/alignments2.php). Each CDR sequence from the IMGT database was compared with the germline CDR sequences of matching length, and the germline CDR sequence with fewest mismatches was assigned as the germline ancestor. The utilization frequency of each germline CDR ancestor in the natural CDR sequence repertoire, as well as the mutation frequency to each of 19 other amino acids at each position of the germline CDR, was analyzed. Synthetic CDR sequences were simulated by selecting germline CDR sequences according to their frequency of occurrence in the natural antibody repertoire and introducing mutations that reflect the natural somatic hypermutation pattern to those germline sequences. The simulation was performed using VBA scripts in Microsoft Excel. Sequences with undesirable PTM motifs were removed from the simulated repertoire. In total, 1,000 sequences for CDR-L2, 7,836 sequences for CDR-H3, and 1,500 sequences for each of the other CDRs were simulated.

### Construction of the library

The simulated CDR sequences with parts of the adjoining framework sequences at 5’ and 3’ ends were synthesized in arrays and stripped from the chip (LC Sciences, Houston, TX, USA). The template scFv genes (DP47-linker-DPK22 and DP47-linker-DPL3) were synthesized by GenScript Inc. (Piscataway, NJ, USA) and cloned to pComb3X and pUC57 vectors. The CDRs were amplified by PCR using specific primer sets (S1 Table). Each amplified CDR was inserted to a template scFv sequence by overlap extension PCR (OE-PCR), and the resulting single-CDR libraries were ligated to pComb3X phagemid vector and transformed to *Escherichia coli* ER2537 as previously described [4]. Phage-displayed scFv libraries were rescued from the transformed *E*. *coli* and subjected to one round of proofreading panning against anti-HA antibody (clone F7; Santa Cruz Biotechnology, Dallas, TX, USA). Specifically, the anti-HA antibody was immobilized on an immunotube (1 μg/mL in 1 mL PBS). After immobilization, the tube was blocked with 3% nonfat dried milk in PBS containing 0.05% Tween 20 (mPBST). Rescued phage library (10^10^ cfu) in 1 mL mPBST was added to the immunotube, incubated at room temperature for 1.5 h, and the tube was washed five times with PBST. The bound phages were eluted with 1 mL of 100 mM triethylamine solution, neutralized with 0.5 mL of 1 M Tris-HCl (pH 7.0), and added to 8.5 mL of mid-log phase ER2537 *E*. *coli*. After incubation for 1 h at 37°C, the infected bacteria were plated on LB-ampicillin agarose plates supplemented with 2% (w/v) glucose. Next day, the bacteria were collected from the plate, and the phagemid vector containing proofread CDR sequences was isolated.

**Fig 1:**
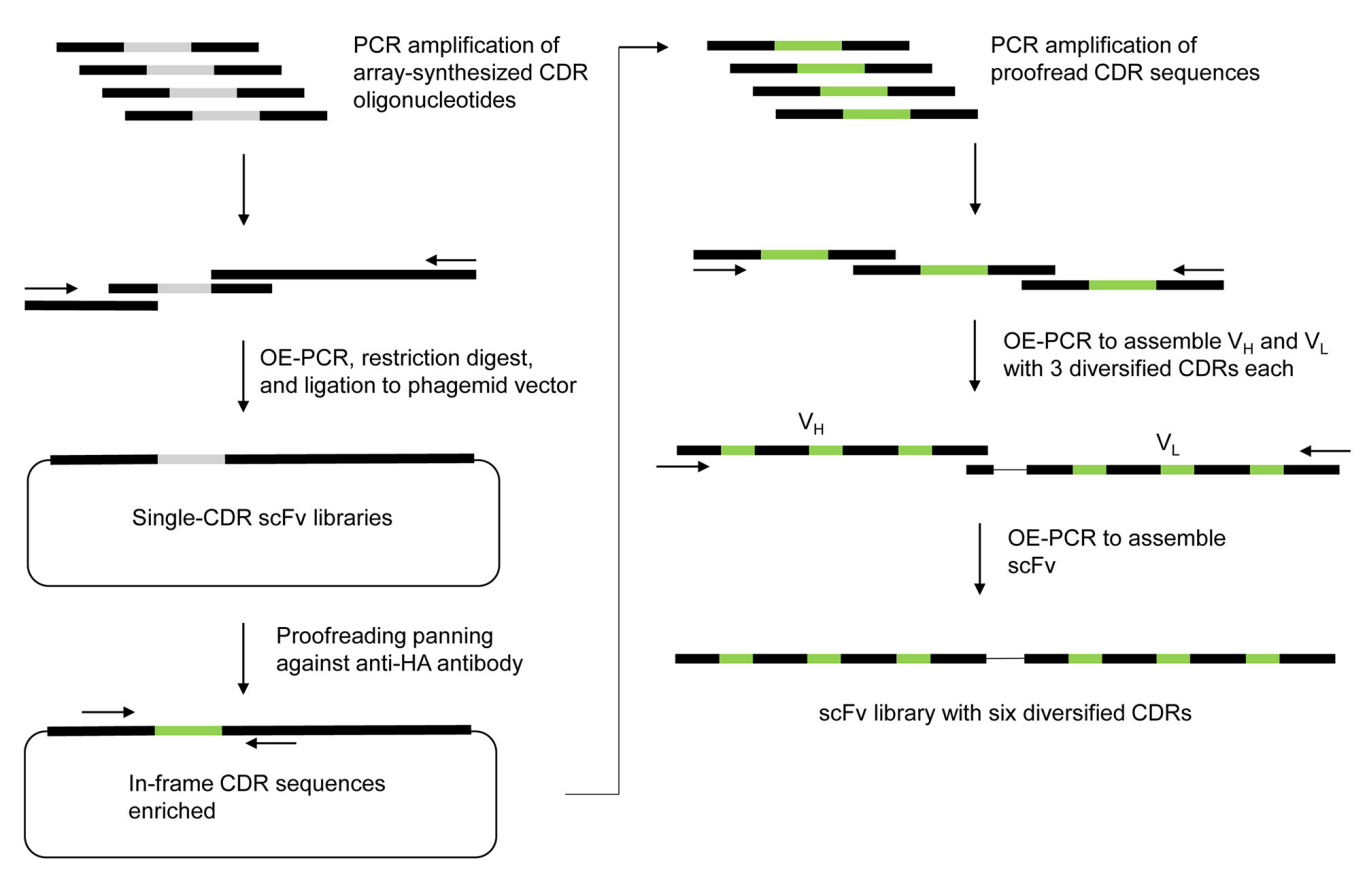
Construction of the scFv library with six non-combinatorially diversified CDRs. Pools of oligonucleotides with the designed CDR sequences were array-synthesized and amplified by PCR. A single-CDR library (scFv library with only one of the six CDRs are diversified) was constructed for each CDR using the amplified CDR oligonucleotide mixtures, and panned against anti-HA antibody that bind to HA-tag at the C-terminus of scFv in order to proofread the CDR repertoires for in-frame sequences. The proofread CDRs were consolidated into the final scFv library with six diversified CDRs.

The proofread CDRs were amplified by PCR, and combined with framework sequences by a series of OE-PCR (Fig 1). The final scFv PCR products with six diversified CDRs were digested with SfiI, ligated to SfiI-digested pComb3X vector, and transformed to ER2537 electrocompetent *E*. *coli* cells. Transformed bacteria were grown overnight in 400 mL of SB media (Super Broth; 3% w/v bactotryptone, 2% w/v yeast extract, and 1% w/v MOPS, pH 7.0) supplemented with 100 μg/mL ampicillin and 2% (w/v) glucose. Next day, the cells were harvested by centrifugation, resuspended in 10 mL SB medium, and frozen in 1 mL aliquots at -80°C after addition of 0.5 volume of 50% glycerol.

### Library panning

Library rescue and panning protocols are described previously [4]. Briefly, one aliquot each of the frozen sub-library *E*. *coli* stocks were grown in 400 mL SB medium with ampicillin and 2% glycerol. When the optical density at 600 nm (OD_600_) reached 0.7, cells were centrifuged, resuspended in 400 mL SB medium with ampicillin, and 10^12^ pfu of VCSM13 helper phage was added. After 1 h incubation at 37°C with gentle shaking, kanamycin (70 μg/mL) was added, and the bacteria were cultured overnight at 30°C. Next day, the cultures were centrifuged and phages were precipitated from the supernatant by adding 4% (w/v) PEG8000 and 3% (w/v) NaCl. Precipitated phages were collected by centrifugation, resuspended in PBS, glycerol was added to 15% final concentration, and frozen in aliquots of 10^13^ cfu at -80°C.

For panning of the library against target molecules, an immunotube was coated with antigen (1~10 μg/mL in PBS) and blocked with mPBST. One aliquot (10^13^ cfu) of the library in mPBST was added to the antigen-coated tube and incubated for 1–2 h. After washing 2–5 times with PBST, bound phages were eluted with triethylamine and neutralized as above. ER2537 *E*. *coli* cells were infected with the eluted phages and grown overnight on LB-ampicillin agar plates with 2% glucose. Next day, cells were harvested and ~10^8^ cells were grown in 20 mL SB medium with ampicillin until OD_600_ reached 0.7, at which point 10^11^ pfu of VCSM13 helper phage was added. After 1 h infection at 37°C with gentle shaking (80 rpm), 70 μg/mL of kanamycin was added and the cells were cultured overnight at 30°C with shaking at 200 rpm. Next day, the culture was centrifuged, and phages were precipitated as described above and used for the subsequent round of panning.

### ELISA screening and dot blot assay

Bacterial colonies from the panning output were grown in SB-ampicillin in 96-well microtiter plates for ~3 h, or until turbid. Isopropyl β-D-1-thiogalactopyranoside (1 mM final concentration) was added to each well, and the plates were incubated overnight with shaking at 30°C. Next day, the plates were centrifuged, supernatants were discarded, and the cell pellets were resuspended in 60 μL of cold 1× TES buffer (20% sucrose, 1 mM EDTA, 50 mM Tris, pH 8.0), and 90 μL of cold 0.2× TES was subsequently added. After incubation on ice for 30 min, the plates were centrifuged and the supernatants containing the periplasmic fraction were added to antigen-coated ELISA plates blocked with mPBST. After 1 h incubation at room temperature, the plates were washed three times with PBST and horseradish peroxidase (HRP)-conjugated anti-HA antibody (clone F-7; Santa Cruz Biotechnology) was added. The plates were incubated for 1 h, washed three times with PBST, and the binding activity was measured using the chromogenic HRP-conjugated substrate tetramethylbenzidine (TMB).

For dot blot assay, random clones from unselected library were grown in a 96-well plate and induced, and periplasmic extracts were obtained as described above. One microliter fractions of the periplasmic extracts were blotted onto nitrocellulose membrane. The membrane was dried and blocked with mPBST and the presence of scFv was probed by enhanced chemiluminescence using HRP-conjugated anti-HA antibody.

### Surface plasmon resonance (SPR)

SPR analysis was performed using a BIAcore 3000 device (GE Healthcare, Piscataway, NJ, USA). Antigen was immobilized on a flow cell of CM5 sensor chip (GE Healthcare) by amine coupling method at approximately 1,000 response units (RUs) following the manufacturer’s protocol. Purified scFv [4] was diluted in degassed PBS at 1–625 nM and injected at a flow rate of 50 μL/minute. Sensogram was obtained at each scFv concentration, and the binding kinetic parameters were evaluated using the BIAevaluation software.

### Next generation sequence analysis of the antibody library

V_H_ and V_L_ sequences of the scFv library were obtained by 300 bp paired-end sequencing on Illumina MiSeq platform. The raw paired-end sequencing data were processed using FLASH [14], and further analyzed by our in-house code implemented in R language. First, framework regions were identified by translating all three reading frames of the sequencing reads and aligning them with 5 amino acid terminal sequences on both ends of the reference framework regions. Point mutations were allowed during the alignment so that framework sequences with errors introduced during synthesis or PCR construction of the library could also be detected. Once each framework region is defined, the sequence between two adjacent framework regions was identified as CDR. The CDR sequences were subsequently analyzed for their identity to the designed CDR sequences and their frequency of occurrence in the library.

## Results

### Analysis of the CDR sequences of natural human antibodies and the simulation of human-like CDR sequences

Human V_H_ and V_L_ sequences were downloaded from the IMGT database (http://imgt.org). Only those sequences for which the end of FR1 and the beginning of FR4 could be identified were selected (so that all three CDRs were included in the sequence), and redundant variable region sequences were removed. In total, 8,846 V_H,_ 3,110 Vκ, and 2,440 V_λ_ sequences were chosen for analysis. CDR sequences were extracted from these variable region sequences [13], and the somatic mutations of the CDRs were identified by comparing their sequences with the closest human germline CDR sequence.

Sequences for CDR-H1, H2, L1, and L2 were then simulated based on the utilization frequency of each ancestral germline CDR sequence in the pool of natural human antibody sequences, and the spectrum of the somatic hypermutations analyzed as described above. For CDR-L3, the first six-to-eight residues (depending on the length of the CDR) were simulated in the same way as described above, and the last two-to-three residues, corresponding approximately to the N-terminal part of the J_L_ segment, were simulated based on the amino acid frequency of the corresponding positions of the actual VJ-recombined human CDR-L3 sequences.

For the analysis of CDR-H3 sequences, the identification of germline ancestors is not practical because VDJ recombination and somatic hypermutation mechanisms produce many sequences that bear little resemblance to the germline D-gene segments. Therefore, CDR-H3 sequences of different lengths were instead analyzed for the frequency of each of 20 amino acids at each position, and these data were utilized to simulate CDR-H3 with different lengths (from 9 to 20 amino acids). The last three residues of CDR-H3 (Kabat numbering 100–102), which typically originate from J_H_ genes and have consensus sequences of Phe/Met—Asp—Tyr/Val/Leu/Ile along with other variants, were analyzed as three amino acid units. The relative frequency of each three amino acid sequence unit in naturally occurring human CDR-H3 was reflected in the design of this part of CDR-H3.

**Fig 2:**
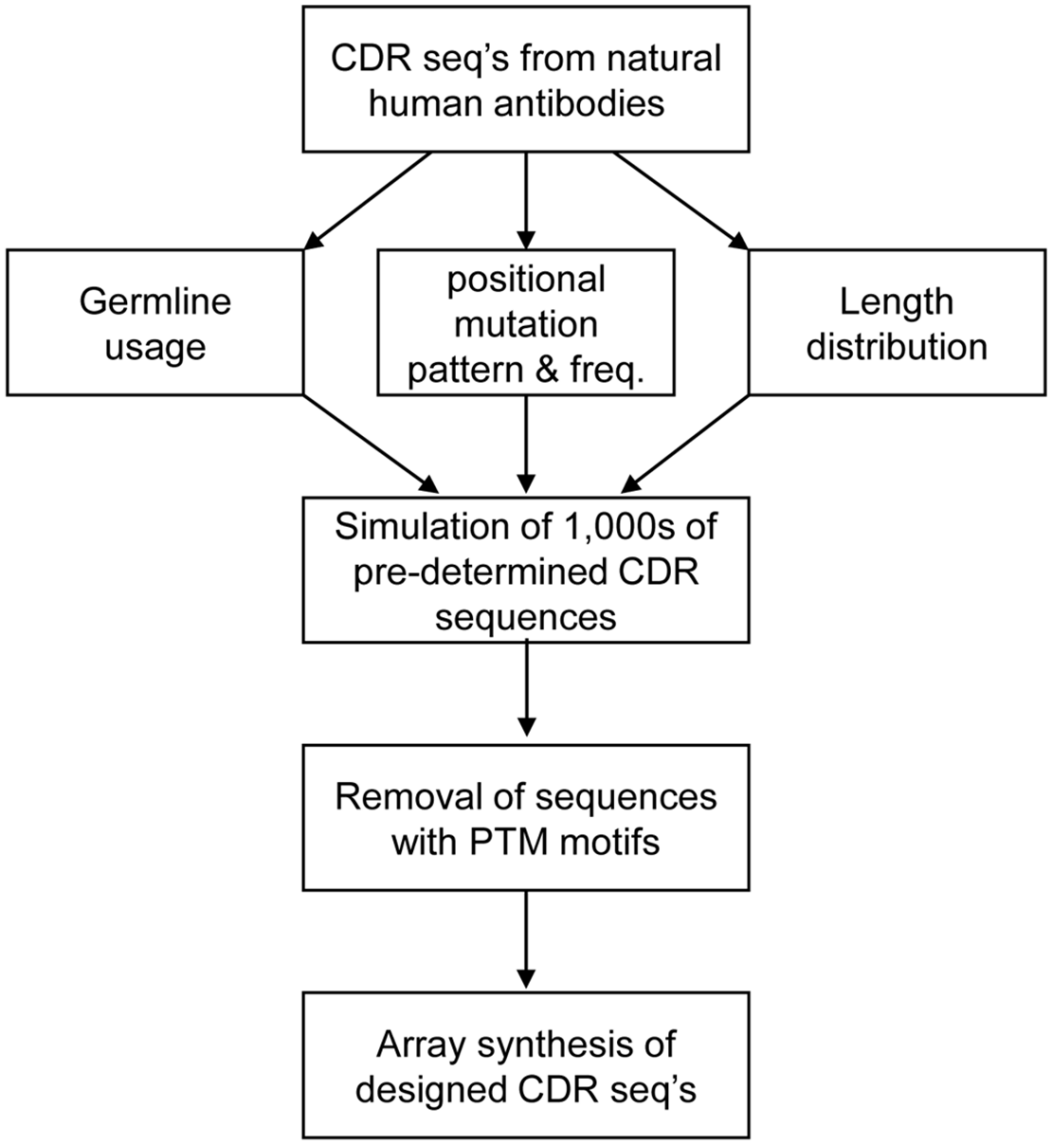
Design process of the non-conbinatorial CDR diversity. CDR sequences of thousands of natural human antibodies were compared with the human germline CDR sequences and their somatic hypermutation patterns/frequency, germline sequence usage, and length distribution were analyzed. Thousands of CDR sequences were simulated based on the analysis and sequences with undesirable post-translational modification motifs were removed from the repertoire. The resulting CDR repertoires contained the sequences that dutifully mimic the naturally produced human CDRs but without many of the deleterious sequence motifs.

After simulation, the CDR sequences were inspected for the presence of potential post-translational modification (PTM) motifs. Specifically, simulated CDR sequences with sites for *N*-glycosylation (NXS/NXT, where X is any amino acid except Pro), deamidation (DG), isomerization (NG), non-enzymatic cleavage (DP), and oxidation (C, M) were removed with the exceptions of H34 of CDR-H1 and H100 of CDR-H3 where methionine is commonly or predominantly found. Some of the germline CDR sequences contain potential PTM sites; these sequences were changed to eliminate the PTM motifs before simulation. For example, human germline CDR-H2 sequences contain many potential deamidation (NG) and isomerization (DG) motifs, and the simulation was performed after substituting these motifs with Ser-Gly. In total, 7,836 of CDR-H3 sequences and 1,000–1,500 each (not counting redundancies) of the other CDRs were designed by the simulation (Table 1). The CDR design process is outlined in Fig 2.

**Table 1:** Number of total and unique CDR sequences designed for the scFv library.

CDR	H1	H2	H3	K1	K2	K3	L1	L2	L3
Total	1,500	1,500	7,836	1,500	1,000	1,500	1,500	1,000	1,500
Unique	502	1,300	7,526	657	202	920	678	279	1,015

### Construction of scFv library with pre-defined CDR sequences

Pools of oligonucleotides encoding the designed CDR sequences, with 5’- and 3’- flanking sequences from the framework regions of human variable heavy chain gene DP-47, kappa light chain gene DPK-22, or lambda light chain gene DPL3, were prepared by array synthesis. A maximum of 3,918 oligonucleotides could be synthesized per pool. Therefore, CDR-H3 (7,836 sequences) were prepared in two separate pools to increase the diversity of this critical region. Other CDRs (a total of 11,000 sequences) were synthesized in three additional pools. These CDR-encoding oligonucleotides were inserted to the template scFv sequence by PCR. After ligation to the phagemid vector pComb3X which has a HA tag at the C terminus of the scFv cloning site and transformation to *E*. *coli* ER2537, these single-CDR libraries were subjected to one round of panning against anti-HA antibody to enrich in-frame CDR sequences. The proofread CDR sequences were amplified and assembled into scFv repertoires with six diversified CDRs by a series of overlap extension PCRs. After ligation and transformation, a final scFv library with a total diversity of ~8×10^8^ individual clones was obtained (Fig 1).

### Sequence analysis

**Fig 3:**
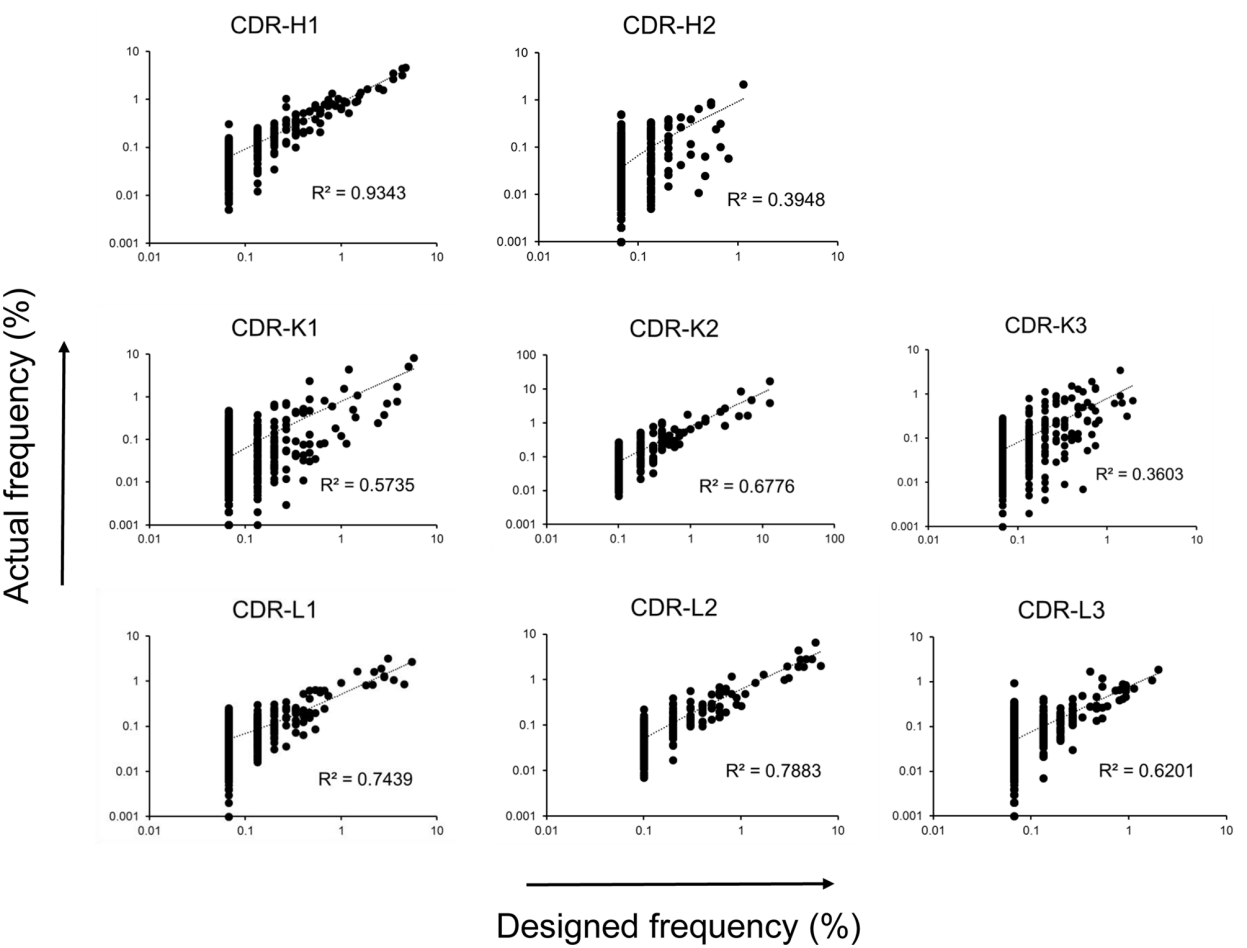
Frequencies of unique CDR sequences in the designed and actual CDR repertoires of the library. The frequencies of occurrence of each unique CDR sequence in the designed and the NGS-analyzed CDR repertoires of the actual scFv library are shown in XY-scatter plots. Each dot in the plot represents a unique CDR sequence. For the NGS-analyzed sequences, only those in the designed repertoires were included in the analysis. CDR-H3 sequences were not analyzed since most of the sequences occur only once in the designed repertoire.

**Fig 4:**
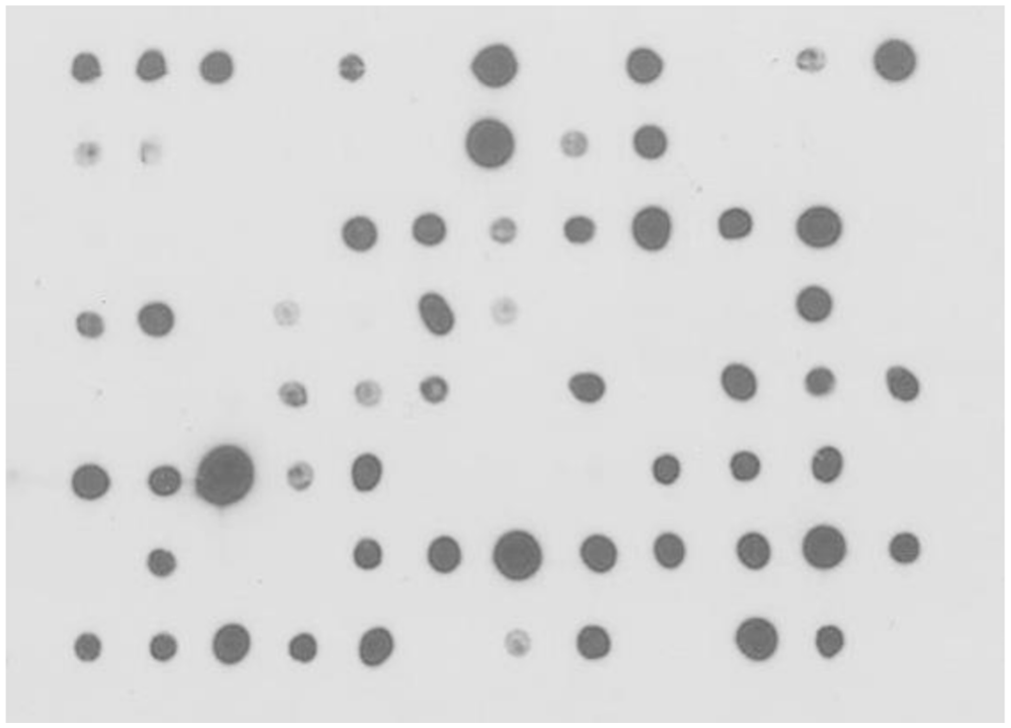
Dot blot analysis of random pre-selection scFv clones from the library. Ninety-two clones were randomly chosen from the unpanned library, grown and induced with IPTG, and periplasmic extracts were obtained and blotted on a nitrocellulose membrane. The clones that express soluble, full-length scFv were detected by the presence of a HA-tag at the C-terminus. Out of 92 clones, 58 were detected by HRP-conjugated anti-HA antibody (63%).

Next generation sequencing (NGS) of the library was performed to assess the fidelity with which the CDR design was reflected in the constructed library. Millions of CDR sequences were analyzed and compared with the designed sequences (Table 2). The designed sequences were nearly completely covered in the constructed library, and the frequency of occurrence of each designed CDR sequence also roughly represented in the actual library (Fig 3), although for long CDRs a majority of the unique sequences occur only once or twice in the designed repertoire and the coefficients of determination (*r* ^2^) are relatively low. Not surprisingly, the library was found to contain many low-frequency CDR sequences not matching any of the designed sequences due to synthesis errors, including non-functional sequences with nucleotide insertions or deletions that cause frameshifts. The proofreading panning of the single-CDR libraries against anti-HA antibody (see above) removed many of the non-functional CDR sequences, and the ratio of functional in-frame CDR sequences was 90–93% compared with 31–86% before the proofreading. An exception was CDR-L2 of the lambda light chain, with only 79% of the sequences in-frame, likely due to inaccurate annealing during the overlap extension PCR. The ratio of CDR sequences with the designed lengths was 82–91% (56% for CDR-λ2). Overall, about 75% of V_H_, V_κ_, and V_λ_ sequences were functional variable domains without stop codon, and the percentage of functional scFv clones in the library was estimated to be approximately 55%. This estimation roughly agreed with the dot-blot assay of randomly chosen library clones. For dot-blot assay, periplasmic extracts of randomly chosen scFv clones from the unselected library were blotted on a nitrocellulose membrane, and the presence of solubly expressed scFv in the extract was probed by detecting the C-terminal HA tag. It was estimated that ~60% of the clones solubly expressed scFv (Fig 4).

**Table 2:** Next-generation sequencing analysis of the library CDRs.

CDR-	H1	H2	H3	K1	K2	K3	L1	L2	L3
Processed sequences (×10^6^)	4.75	4.70	4.53	2.58	2.31	2.52	2.14	2.12	2.09
In frame %	91.3	89.7	90.3	92.2	91.6	93.2	91.5	78.7	90.7
Designed length %	89.4	88.4	85.6	87.1	82.3	90.7	84.6	56.2	89.4
Designed sequence %	80.9	52.0	51.8	62.4	70.3	74.3	56.2	47.6	67.2
In frame % before proofreading^a^	85.7	50.0	67.9	53.3	44.4	66.7	38.5	31.3	66.7
Design coverage %^b^	100	100	97.3	99.8	100	99.9	100	100	100

^a^ Percentage of in-frame CDR sequences before the proofreading panning was estimated by Sanger sequencing of 12~28 sequences per CDR.

^b^ Percentage of the designed CDR sequences that were found in NGS analysis.

**Fig 5:**
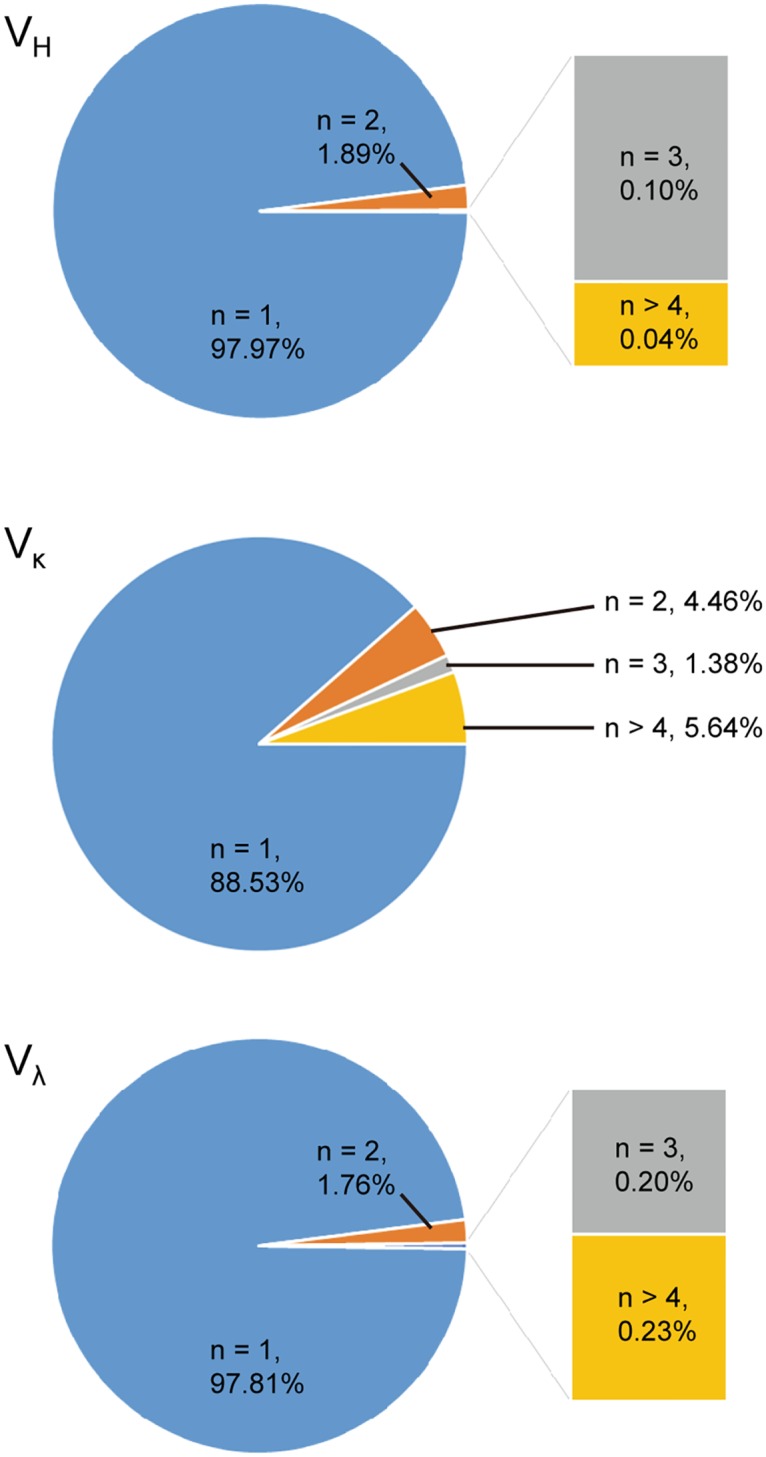
Variable domain sequence redundancies of the constructed library. The variable domain sequences of the unselected library were obtained by 300 bp paired-end sequencing on Illumina MiSeq platform, and the number of replicates (n) for the variable domain sequences was analyzed. Approximately 98% of V_H_ and V_λ_, and 88.5% of V_κ_ sequences were found only once (n = 1).

The uniqueness of the variable domains was analyzed from the NGS data. When ~1.3 million each of the heavy, kappa, and lambda variable domain sequences without stop codons were analyzed, 98% of V_H_, 89% of V_κ_, and 98% of V_λ_ sequences were non-redundant (Fig 5), and the percentages of the number of different variable domain sequences among total sequence reads were 99%, 92%, and 99% for V_H_, V_κ_, and V_λ_, respectively. These values are comparable to the CDR-H3 sequence uniqueness of 97~98% for other highly diverse antibody libraries [9, 15], and suggest that the redundancy among scFv clones in the unselected library is not significant.

**Fig 6:**
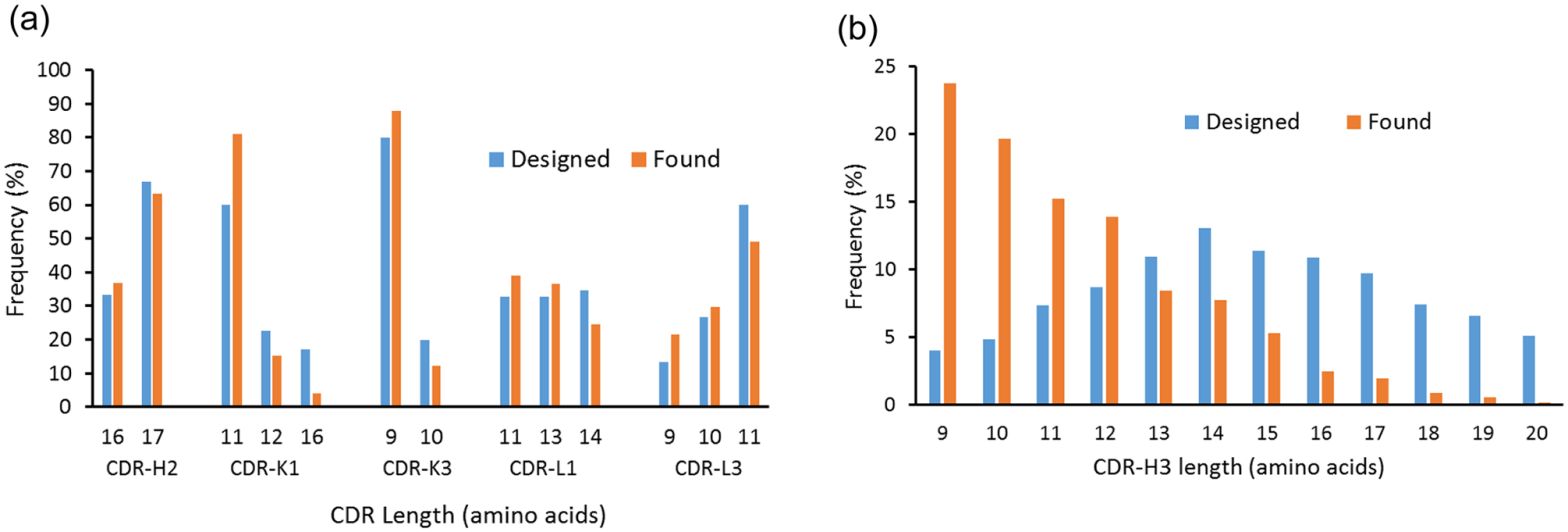
The length distribution of the designed and the actual CDRs. (a) CDR-H2, L1 (kappa and lambda), and L3 (kappa and lambda) contain sequences with varying lengths. The length distribution of the designed repertoire and the NGS analysis results suggest that shorter CDRs are preferentially incorporated in the constructed library. (b) The preference for the shorter CDRs was more evident in CDR-H3 which has a wider range of length variation than other CDRs. In both (a) and (b), the blue bars (“Designed”) indicate the frequency of each CDR length in the designed repertoire, and the orange bars (“Found”) indicate the frequency of each CDR length found from the NGS analysis of the constructed library. See text for details.

The distribution of CDR length, especially of CDR-H3 length, in the constructed library differed from the design, with shorter length CDRs conspicuously overrepresented when compared to longer CDRs (Fig 6). This is probably in part because of the inaccuracy during the oligonucleotide array synthesis that introduced frameshifts and premature stop codons. Because these errors are more likely to occur during the synthesis of longer CDRs and most of them would be removed during the proofreading panning of the single-CDR libraries against anti-HA-tag antibody (see above), it is plausible that more of the longer CDRs were removed from the library. Also conceivable is the possibility that scFvs with shorter CDRs were preferentially enriched by the panning against anti-HA-tag antibody [16].

**Fig 7:**
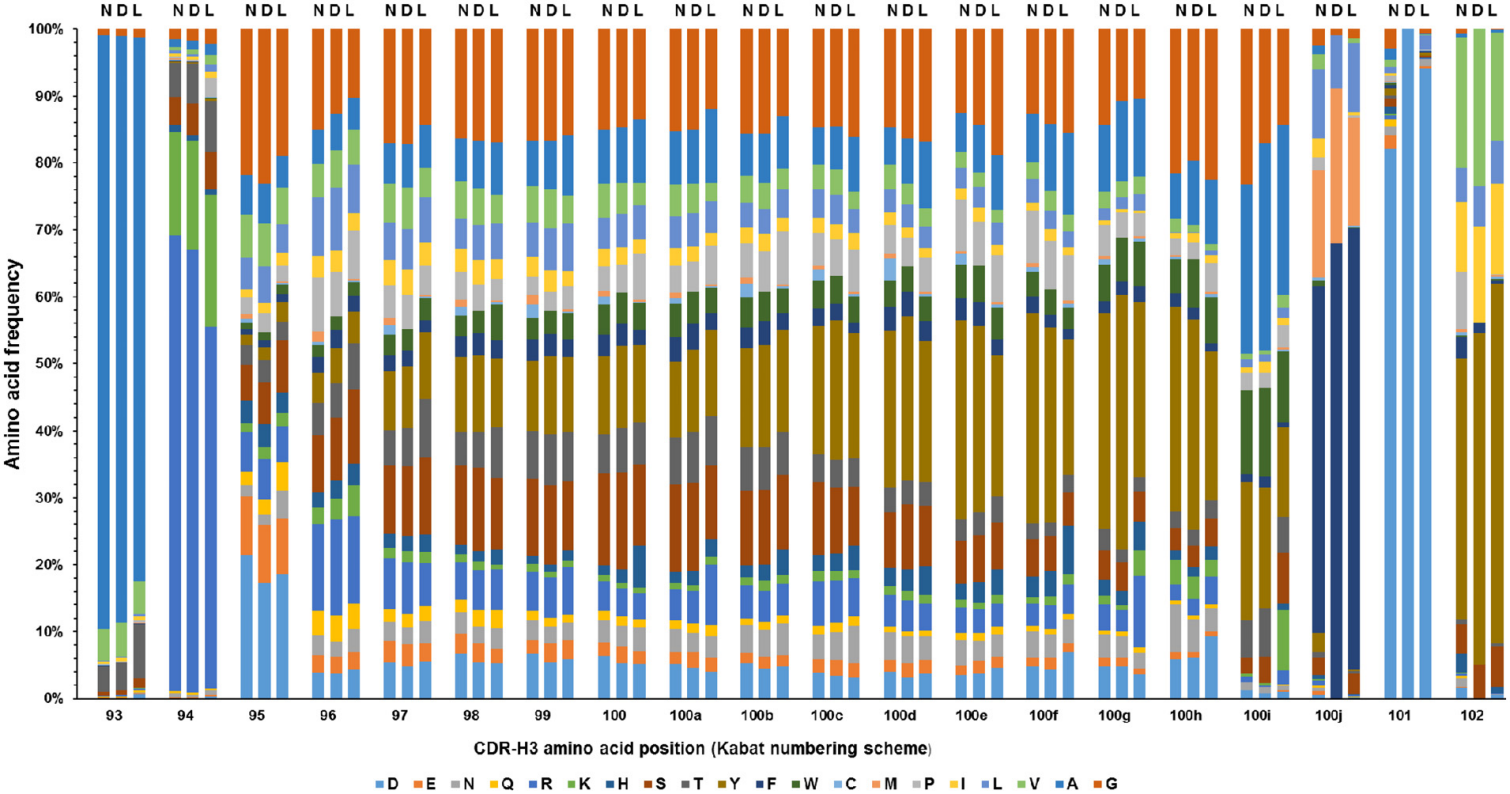
The CDR-H3 amino acid distributions. The amino acid distributions of the CDR-H3 of natural human antibodies (N), the designed repertoire (D), and the actual constructed library (L) are shown for each position. Each stacked bar reflects the combined frequencies of amino acids at each Kabat position of CDR-H3 of different lengths. For all CDR-H3s with different lengths, the last three residues are denoted by 100j, 101, and 102, respectively.

The similarity of the library CDR sequences to the natural CDR sequences was assessed by analyzing the number of amino acid differences in each CDR sequence from the closest germline CDR sequence. Because the CDRs were designed to simulate the natural SHM patterns, it was assumed that the designed sequences are highly nature-like. Indeed, the average numbers of mutations per CDR sequence were comparable between the designed and the natural CDR sequences (Table 3). When the library CDR sequences that did not match any of the designed sequences (due to synthesis errors) were analyzed, the average number of amino acid differences from the closest germline CDR sequence was different from that of the designed sequences by only 1–2 amino acids on average. These results suggest that the CDR sequences of the library contain only small numbers of mutations from the human germline CDR sequences, and are highly similar to the CDR sequences of natural human antibodies. For CDR-H3, the amino acid distribution at each position was analyzed (Fig 7). Highly similar distribution patterns were found among the CDR-H3s of natural human antibodies, the simulated repertoire, and the constructed library, further demonstrating the nature-likeness of the library CDRs.

**Table 3:** Average numbers of amino acid differences in the CDR sequences from the closest human germline CDR sequence.

CDR^a^	H1	H2		K1			K2	K3		L1			L2	L3		
Length	5	16	17	11	12	16	7	9	10	11	13	14	7	9	10	11
Designed^b^	0.75	2.14	2.55	1.00	1.18	1.85	0.47	0.73	0.65	1.03	1.10	0.98	0.61	0.83	1.18	0.70
Non-designed^c^	1.84	4.30	3.67	2.77	3.44	3.82	2.01	1.55	1.45	2.82	3.22	2.75	1.85	1.68	2.11	1.45
Natural^d^	0.82	2.09	2.37	1.08	1.29	0.93	0.49	0.72	0.54	0.95	1.10	1.00	0.66	0.88	1.14	0.75

^a^ CDR-H3 was not analyzed because there were no germline sequences to compare with.

^b^ Designed CDR sequences as described in the text.

^c^ The library CDR sequences that did not match any of the designed sequences but had the correct length.

^d^ Natural human CDR sequences with corresponding length retrieved from the IMGT database.

As expected, the occurrence of undesirable PTM motifs in the CDRs was much lower than in natural human antibody CDRs, with the exceptions of CDR-H1 and CDR-L2, which are short (5 and 7 amino acids, respectively) and have relatively few PTM motifs in natural human antibodies (Table 4). Assuming that the PTM motifs occur independently in different CDRs, the probability of at least one PTM motifs occurring in a scFv sequence in the library was estimated to be 20–30% (70–80% of the clones without PTM motifs), whereas only between 24 to 27% of scFvs from natural sources would be free of the PTM motifs.

**Table 4:** Percentages of post-translational modification motifs in the CDRs of the library and the natural human antibodies.

Motif	CDR-	H1	H2	H3	K1	K2	K3	L1	L2	L3
Asp-Gly	library	0.05	0.31	0.66	0.03	0.34	0.10	0.32	3.41	0.34
	natural	0.08	10.79	6.45	6.03	0.32	0.09	0.28	0.30	1.98
Asn-Gly	library	0.04	0.62	0.32	0.05	0.63	0.12	0.60	4.51	0.32
	natural	0.02	5.96	3.03	4.54	0.53	0.50	0.06	0.12	7.61
Asp-Pro	library	0.01	0.16	0.25	0.04	0.08	0.10	0.04	0.20	0.05
	natural	0.00	2.40	10.13	0.00	0.04	0.14	0.11	0.00	0.66
N-glyc.	library	0.66	1.91	2.77	1.35	1.46	1.00	1.97	0.94	1.30
	natural	0.59	0.78	7.73	0.61	0.07	1.69	6.74	0.30	8.20
Met	library	0.35	1.03	1.80	0.90	0.55	0.29	1.78	0.70	1.70
	natural	0.06	2.72	9.04	0.88	0.11	12.36	0.39	0.18	0.86
Cys	library	0.30	1.96	0.49	1.30	0.64	0.77	2.10	0.87	1.29
	natural	0.35	17.50	1.46	0.92	0.18	1.19	0.61	0.24	1.98
Total PTM	library	1.36	5.44	5.87	3.60	3.58	2.32	6.16	10.48	4.81
	natural	1.09	36.70	32.92	12.55	1.23	15.56	8.08	1.13	20.70

### Panning and screening of the library on antigens

The constructed library was panned against four protein antigens to validate its functionality. Multiple target-binding scFv clones were isolated from the library after four rounds of panning on antigens passively adsorbed on the plastic surface. Output colonies from the third or fourth round of panning were screened by ELISA, and some of the clones with positive signal were sequenced (Table 5). Interestingly, a majority of the isolated clones were of lambda light chain class, although the number of the antigens tested and the clones sequenced were not enough for the generalization of the light chain class preference. The preferential selection of clones with specific light chain family/class from phage antibody libraries has been reported previously [17] and has been attributed to the preferential pairing of specific V_H_-V_L_ domains and the difference in the functional sizes of the sub-libraries. Strong preference for lambda light chain after antigen-driven phage display selection of large natural scFv libraries [16, 18] has also been reported, suggesting that the lambda chain preference may be a more-or-less universal phenomenon of the phage display selection of scFv libraries, rather than a characteristic of a specific antibody library. It is noted that the preference for lambda light chain may be dependent on the format of the antibody fragment displayed and restricted to scFv format, as Tiller et al. [15] reported similar levels of display for Fabs with kappa and lambda light chains.

**Table 5:** Panning and screening of the library against antigens.

Antigen	Number of rounds	ELISA positive/screened	Unique sequences/Total sequenced	Kappa/lambda^a^
AIMP1	3	10/94	6/6	1/5
SerRS	4	16/94	3/14	0/14
hEpCAM-ECD	4	18/94	3/15	3/12
HER3-ECD	4	46/188	6/16	8/8
HEW Lysozyme	4	151/188	6/13	1/12

^a^ Numbers of scFvs with kappa or lambda light chain among the sequenced clones.

Binding kinetics of some of the ELISA-positive scFv clones isolated from the library were analyzed by SPR (Table 6). Dissociation constants (*K*_d_) ranging from 10^−9^ to 10^−7^ M were obtained for 10 scFvs against three different antigens. These values were comparable to those from other previously reported antibody libraries [4, 7, 9] as well as typical hybridoma-derived antibodies [19, 20], supporting the validity of the non-combinatorial CDR design approach.

**Table 6:** Binding kinetics of selected target-specific scFv clones determined by SPR.

Antigen-clone	*k*_on_ (M^-1^s^-1^)	*k*_off_ (s^-1^)	*K*_D_ (M)
HER3-G3	6.8 × 10^5^	2.8 × 10^−3^	4.2 × 10^−9^
HER3-C5	1.2 × 10^3^	1.0 × 10^−3^	8.1 × 10^−9^
HER3-D11	7.8 × 10^4^	5.3 × 10^−3^	6.8 × 10^−8^
HEWL-B6	1.1 × 10^5^	5.3 × 10^−3^	4.8 × 10^−8^
HEWL-F8	5.8 × 10^4^	1.9 × 10^−3^	3.3 × 10^−8^
HEWL-B2	1.1 × 10^6^	3.0 × 10^−3^	2.8 × 10^−9^
HEWL-G7	1.4 × 10^5^	3.2 × 10^−3^	2.4 × 10^−8^
SRS-A3	2.7 × 10^4^	1.8 × 10^−3^	6.7 × 10^−8^
SRS-C4	9.5 × 10^4^	1.3 × 10^−3^	1.4 × 10^−8^
SRS-D8	6.5 × 10^4^	1.4 × 10^−3^	2.2 × 10^−8^

The NGS results from the unselected library were compared with the sequences of the unique scFvs selected from the library after panning. The panning process did not appear to significantly alter the percentage of the designed CDR sequences or the average number of mutations per CDR residue (Table 7), suggesting that the additional CDR diversity introduced by the errors in oligonucleotide synthesis or PCR was not critical to the performance of the library. On the other hand, the mutation frequency in framework regions (FRs) decreased after the panning selection (Table 7). It is plausible that some of the mutations in the FRs had deleterious effects on the folding, stability, expression, and/or display of scFv and were selected against during the panning.

**Table 7:** Mutations in CDRs and FRs before and after the panning selection.

CDR or FR	H1	H2	H3	K1	K2	K3	L1	L2	L3	V_H_ FR	V_κ_ FR	V_λ_ FR
% designed, unselected^a^	80.9	52.0	51.8	62.4	70.3	74.3	56.2	47.6	67.2			
% designed, selected^a^	92.0^c^	48.3^c^	64.5^c^	57.1^c^	60.0^c^	83.3^c^	60.0^c^	60.0^c^	60.0^c^			
mutation/residue, unselected^b^	0.21	0.18	n/a^e^	0.13	0.11	0.06	0.11	0.12	0.13	0.0067	0.0144	0.0187
mutation/residue, selected^b^	0.17^c^	0.17^c^	n/a^e^	0.14^c^	0.10^c^	0.09^c^	0.14^c^	0.11^c^	0.10^c^	0.0018^d^	0.0019^d^	0.0046^d^

^a^ Percentage of the designed CDR sequences in total unselected or selected CDR sequences.

^b^ Average number of mutations from the closest germline CDR sequence or the reference framework sequence per amino acid position.

^c^ *P* > 0.15 between the values for selected and unselected CDR sequence.

^d^ *P* < 0.05 between the values for selected and unselected FR sequences.

^e^ Designed CDR-H3 sequences have no germline ancestors.

## Discussion

Unlike natural antibodies, the synthetic antibody diversity is generated by the concatenation of random mono- or trinucleotide units [2–7]. Very large sequence diversity can be easily prepared this way, and a number of sophisticated synthetic approaches have been employed to produce sequences that resemble natural CDRs [9, 15]. However, there are intrinsically uncontrollable aspects in the process of random nucleotide synthesis. Even when a CDR is designed to contain only those amino acids that occur frequently at each position, not all of the resulting individual CDR sequences will be nature-like because the combination of the nature-like residues at each position may result in sequences that are unlikely to be found in natural antibodies. For example, the combination of the most frequently occurring residues at the human variable heavy chain CDR-H2 positions 50_H_-56_H_ is WISPDGG, which is not found in known human antibody sequences. The less nature-like CDR sequences may adversely affect the folding and stability of the antibody as well as increasing immunogenicity when administered to human patients.

Both in natural and synthetic antibody repertoires, sequences with undesirable PTM motifs are frequently found in the variable regions. For example, the *N*-glycosylation motif (N-X-S/T) is frequently found in the CDRs of antibodies. Although human germline variable genes contain few *N*-glycosylation sequences, SHM introduces many of them to mature antibodies. Likewise, many synthetic antibodies also have these motifs in their CDRs. This is partly because Asn, Ser, and Thr are among the more frequently utilized amino acids in the antibody CDRs [11, 12]. The sugar moiety is structurally heterogeneous and host-dependent, and may interfere with target binding activity of the antibody. Also, when present in the antibody library, these motifs are not glycosylated during the amplification phase, which is usually performed using a prokaryotic host or *in vitro* translation system, often resulting in the loss of the antibody binding activity upon expression from eukaryotic host.

Other modifications, such as deamidation of Asn residues [21, 22], isomerization of Asp residues [21, 22], peptide backbone cleavage [23], and amino acid side chain oxidation [21], occur spontaneously, non-enzymatically and relatively slowly after secretion from the host cells. The reaction rates for the deamidation of asparagine and the isomerization of aspartate are dependent on the sequence context, and especially on the amino acid that immediately follows these residues [21, 22]: the reactions proceed significantly faster when Asp or Asn residues are followed by a glycine. Therefore Asp-Gly and Asn-Gly sequences were completely eliminated from the CDRs and CDR-framework region (FR) junctions. While other Asn and Asp-containing sequences can also be modified by deamidation and isomerization, respectively, at slower rates, they were too numerous to be avoided and were included in the final library design. Peptide backbone cleavage can lead to protein fragmentation and loss of the functional activity of the protein upon prolonged storage, especially at acidic or basic conditions. The Asp-Pro sequence is particularly prone to such fragmentation [23], and while this degradation reaction is reportedly negligible at neutral or slightly acidic pH, the rarely occurring Asp-Pro sequences were also removed from the CDR design. Lastly, the side chains of a number of amino acids can undergo oxidation, of which sulfur-containing amino acids cysteine and methionine are most prominent. Therefore, these residues were also removed from the CDR design, except for two positions (H34 of CDR-H1 and H100 of CDR-H3) at which methionine is commonly or predominantly found in natural antibodies.

A pioneering example of the PTM site avoidance in an antibody library has been reported previously [15]. The Fab library by Tiller et al. contains non-randomized, germline-based CDR1s and 2s with carefully introduced mutations to eliminate the PTM sites, and PTM-prone amino acids such as Met, Cys, Asn, and Asp were removed or minimized in CDR3s. Compared with this approach, our predefined, non-combinatorial CDR design approach allows greater diversity of CDR1s and 2s, but CDR sequences with different germline origins are grafted onto a single framework, potentially introducing unnatural CDR-FR junction sequences. The natural abundances of Asp and Asn in CDR3s are also maintained in our library, while the much lower diversity in CDR3s may have a negative effect on the performance of the library (see below).

Approximately 60% of randomly selected clones from the library were observed to express soluble scFv by dot blot assay. This is consistent with the sequencing data from which it was estimated that the library contained about 55% of functional scFv clones (calculated from the percentages of in-frame CDR sequences in Table 2). As expected, the occurrence of PTM motifs in CDRs was lower than that of natural human antibodies. Although the library was originally designed to be devoid of PTM sequences, synthesis errors introduced CDR sequences with undesirable motifs. Synthesis errors also likely biased the CDR length distribution, resulting in the enrichment of short CDR sequences. Sub-stoichiometric coupling efficiency during oligonucleotide synthesis produces shorter and/or out-of-frame CDR sequences. Because these errors are more likely to occur in longer CDRs and most of them would be removed by the proofreading panning against anti-HA antibody, shorter CDRs are relatively more enriched over longer ones in the final library. The biasing effect of the sub-stoichiometric coupling efficiency on the CDR length distribution has been reported previously [7], in which CDRs synthesized by trinucleotide mutagenesis technology showed a marked shift of the length distribution of CDR-H3 toward shorter sequences. Additionally, the proofreading panning process itself may have caused the preferential selection of clones with short CDRs. In one previous example, the distribution of CDR-H3 length of a naïve human scFv library [16] shifted slightly toward shorter length after antigen-driven selection. Because the proofreading panning selects for the level of scFv display on phage surface and not for the binding of scFv to the antigen, it is conceivable that the panning against anti-HA antibody had a greater effect on CDR length, which in turn affected the folding and stability of the antibody.

It is noteworthy that despite the low diversity of each CDR, and especially of CDR-H3 which plays a disproportionately large role in majority cases of antibody-antigen interaction [24], multiple clones with nanomolar affinity to their targets were isolated from the library. Antibody libraries with limited diversity in CDR-H3 have been reported previously [25–28]. In one such report, Persson et al. [28] demonstrated that a library with fixed CDR-H3 and diversified CDR-L3 can yield target-specific antibody clones. In another example [27], a library with tetranomial CDR diversity was successfully panned against VEGF to generate high-affinity binders, suggesting that antigen binding sites can be formed of highly limited chemical diversity. Nonetheless, these libraries still rely on random combinatorial events during the synthesis of the oligonucleotides encoding CDRs and the number of possible codon combinations can be quite large. Our results confirm that the antibody library does not necessarily have CDRs with large diversity derived from random combination at the nucleotide or codon level, and that CDRs with pre-defined, non-combinatorial sequence diversity can be combined to generate a highly functional antibody library. The functionality of our library as well as the libraries with restricted CDR-H3 diversity also suggest that the total diversity of antigen combining site is more important than CDR-H3 diversity, and the relative importance of CDR-H3 in antigen binding is probably more of a result, rather than a cause, of the evolution of the large CDR-H3 diversity by VDJ recombination. On the other hand, it also needs to be noted that the enrichment of the binders against the standard antigens such as lysozyme and EpCAM seemed to be slower than other libraries of comparable diversity [3, 4, 29] and as many as four rounds of panning were required to obtain moderate numbers of binders. It is conceivable that the limited diversity of the CDRs and the relative scarcity of mid- to long-length CDR-H3 (Fig 6b) may have contributed to this phenomenon, although further testing is needed to confirm the efficiency of binder enrichment for this library.

To summarize, a semi-synthetic human scFv library with pre-defined synthetic diversity in all six CDRs was designed and constructed. Each CDR has a low diversity of fewer than 10,000 unique sequences, and was designed to mimic natural CDRs with somatic hypermutation but without some of the undesirable PTM motifs. The resulting library with approximately 10^9^ individual clones was successfully panned against a number of antigens and yielded multiple clones with dissociation constants in nanomolar range. The strategy of combining multiple regions of low, pre-defined diversity into a single large protein sequence repertoire has several advantages that include minimizing undesirable sequences and more dutifully emulating naturally occurring proteins, and provides a novel approach to the design of antibody library as well as other alternative scaffold protein libraries.

## Supporting Information

S1 Table Primer sequences used for the construction of the library. (DOCX)
