# Supplementary material for: A Novel Human scFv Library with Non-Combinatorial Synthetic CDR Diversity
Source: PLoS One. 2015 Oct 20;10(10):e0141045. doi: 10.1371/journal.pone.0141045 (PMC4613135; doi:10.1371/journal.pone.0141045)
Supplement: S1 Table — (DOCX) [file pone.0141045.s001.docx]

S1 Table. List of primers used for the construction of the library.

| Primer | Sequence | Note |
| --- | --- | --- |
| H1-f^*^ | CAGCGGATTCACCTTCAGC | Amplification of CDR-H1 |
| H1-b^*^ | AGGTGCTTGGCGAACCCA |  |
| H2-f^*^ | ggcctggaatgggtgagc | Amplification of CDR-H2 |
| H2-b^*^ | GCGGCTGATGGTAAAGCG |  |
| H3-f^*^ | ggacaccgcagtctactact | Amplification of CDR-H3 |
| H3-b^*^ | CACCAGAGTACCTTGTCCC |  |
| K1-f^*^ | cgcgcaacactgtcatgc | Amplification of CDR-L1 of kappa LC class |
| K1-b^*^ | TGGAGCCTGACCTGGTTTC |  |
| K2-f^*^ | ccaggtcaggctccacgt | Amplification of CDR-L2 of kappa LC class |
| K2-b^*^ | ACCGCTTCCAGATCCTGAG |  |
| K3-f^*^ | ctggaacctgaggactttg | Amplification of CDR-L3 of kappa LC class |
| K3-b^*^ | ACTTTAGTGCCCTGACCG |  |
| L1-f^*^ | gcgcgtgactattagctgt | Amplification of CDR-L1 of lambda LC class |
| L1-b^*^ | AGGTGCAGTTCCAGGCAGT |  |
| L2-f^*^ | gcctggaactgcacctaag | Amplification of CDR-L2 of lambda LC class |
| L2-b^*^ | GCCTGATTTGCTACCGCTA |  |
| L3-f^*^ | cttcgctccgaagatgaag | Amplification of CDR-L3 of lambda LC class |
| L3-b^*^ | GTCAGCTTGGTACCGCCA |  |
| JH-f^*^ | ctggtgaccgtgagcagc | Amplification of the linker region and VH (rc) |
| kFR1-f^*^ | GAAATCGTGCTGACCCAG | Amplification of Vκ or the linker region (rc) |
| lFR3-f* | CTGGCCATCAGCGGCCTTC | Amplification of Vλ or the linker region (rc) |
| pC3X-f | GCACGACAGGTTTCCCGAC | Forward primer for VH amplification |
| pC3X-b | AACCATCGATAGCAGCACCG | Reverse primer for VL amplification |
| pC3-seq | GTGAGCGGATAACAATTGA | Forward primer for scFv amplification |
| dp-seq | AGAAGCGTAGTCCGGAACG | Reverse primer for scFv amplification |

* Reverse-complement (rc) sequences of these primers were also prepared and used for the amplification of the proofread CDRs.
